# Polyphenol Oxidases in Crops: Biochemical, Physiological and Genetic Aspects

**DOI:** 10.3390/ijms18020377

**Published:** 2017-02-10

**Authors:** Francesca Taranto, Antonella Pasqualone, Giacomo Mangini, Pasquale Tripodi, Monica Marilena Miazzi, Stefano Pavan, Cinzia Montemurro

**Affiliations:** 1 SINAGRI S.r.l.-Spin off dell’Università degli Studi di Bari “Aldo Moro”, 70126 Bari, Italy; cinzia.montemurro@uniba.it; 2 Dipartimento di Scienze del Suolo, della Pianta e degli Alimenti, Università degli Studi di Bari “Aldo Moro”, 70126 Bari, Italy; antonella.pasqualone@uniba.it (A.P.); giacomo.mangini@uniba.it (G.M.); monicamarilena.miazzi@uniba.it (M.M.M.); stefano.pavan@uniba.it (S.P.); 3 Consiglio per la ricerca in agricoltura e l’analisi dell’economia agraria, Centro di ricerca per l’orticoltura, 84098 Pontecagnano Faiano, Italy; pasquale.tripodi@crea.gov.it

**Keywords:** browning, polyphenol oxidase activity, *PPO* genes, *Solanaceae*, *Poaceae*

## Abstract

Enzymatic browning is a colour reaction occurring in plants, including cereals, fruit and horticultural crops, due to oxidation during postharvest processing and storage. This has a negative impact on the colour, flavour, nutritional properties and shelf life of food products. Browning is usually caused by polyphenol oxidases (PPOs), following cell damage caused by senescence, wounding and the attack of pests and pathogens. Several studies indicated that PPOs play a role in plant immunity, and emerging evidence suggested that PPOs might also be involved in other physiological processes. Genomic investigations ultimately led to the isolation of PPO homologs in several crops, which will be possibly characterized at the functional level in the near future. Here, focusing on the botanic families of *Poaceae* and *Solanaceae*, we provide an overview on available scientific literature on PPOs, resulting in useful information on biochemical, physiological and genetic aspects.

## 1. Introduction

Polyphenol oxidases (PPOs) are a group of Cu-containing enzymes that catalyse the oxidation of several phenols to *o*-quinones [1,2]. In turn, *o*-quinones are highly reactive molecules that can undergo non-enzymatic secondary reactions to form brown complex polymers known as melanins and cross-linked polymers with protein functional groups [3,4,5,6].

In plants, PPOs are nearly ubiquitous and located in chloroplasts. The loss of sub-cellular compartmentalization, due to senescence, wounding, interactions with pests and pathogens and handling during postharvest processing and storage, results in contact between PPOs and vacuolar phenolic substrates [7]. This ultimately leads to a reaction known as enzymatic browning, negatively affecting colour, flavour, nutritional properties and shelf life of food products. However, in a few cases, enzymatic browning might be beneficial, as it leads to the formation of compounds conferring characteristic flavours [8].

Due to the economic importance of browning, PPOs have been extensively studied in relation to their physico-chemical properties. In contrast, knowledge on role of these enzymes in plant physiology is still limited and will possibly increase by the functional characterization of PPO homologs that are now being identified in several crop species. In this review, we take advantage of recent scientific literature to provide an overview on biochemical, physiological and genetic aspects of PPOs. Focus is given to the well-studied PPOs from the botanic families of *Solanaceae* and *Poaceae*. Few highlights in other main crops are also discussed.

## 2. Polyphenol Oxidases (PPO) and Browning

### 2.1. PPO Biochemical Properties

**Figure 1:**
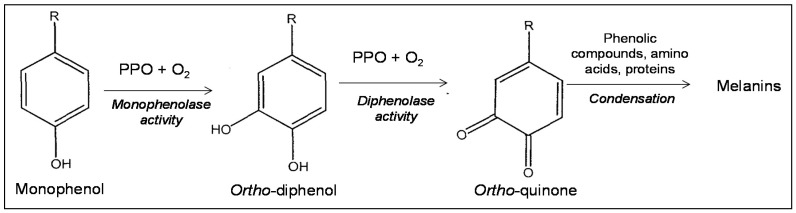
Simplified schematization of browning process. PPO, polyphenol oxidases.

The conversion of phenolic substrates to *o*-quinones by PPOs occurs by means of two oxidation steps. The first is the hydroxylation of the *ortho*-position adjacent to an existing hydroxyl group (“monophenol oxidase” or “monophenolase” activity, also referred to as hydroxylase or cresolase activity). The second is the oxidation of *o*-dihydroxybenzenes to *o*-benzoquinones (“diphenol oxidase” or “diphenolase activity”, also referred to as catecholase or oxidase activity) (Figure 1) [9,10]. Accordingly, PPOs correspond to the following sub-classes indicated by the Nomenclature Committee of the International Union of Biochemistry and Molecular Biology (NC-IUBMB): (i) monophenol monooxygenases (E.C. 1.14.18.1); and (ii) enzymes catalysing the oxidation of diphenols using oxygen as electron acceptor (subclass E.C. 1.10.3.), including catechol oxidases (E.C. 1.10.3.1) and laccases (E.C. 1.10.3.2).

Based on the specific substrate and their mechanism of action, PPOs are classified in three different types: tyrosinases, cathecol oxidases and laccases. Tyrosinases have both cresolase and catecholase activities [11]. Catechol oxidases, also known as *o*-diphenol oxidases, catalyse the oxidation of *o*-diphenols to *o*-quinones. Finally, laccases are capable to oxidize a wide spectrum of aromatic compounds by a radical catalysed reaction mechanism [12].

PPOs have been extensively studied with respect to physico-chemical properties [10,11,12,13] and PPO isoenzymes can be distinguished based on electrophoretic mobility, optimal temperature and pH and substrate specificity [14,15,16,17]. PPO activity depends on the pH, which affects the binding of substrates and the catalysis [18,19,20]. Generally, the optimal pH of PPOs ranges between 4.0 and 8.0. In several Prunoideae (almond, apricot, peach, and plum), PPOs exhibit maximal activities around pH 5.0 [21]. Similarly, apple PPOs have an optimal pH from 4.5 to 5.0 [22]. Cherry and strawberry PPOs show a narrow pH optimum at about 4.5 [21,22,23]. PPOs from pineapple, having (+)-catechin as substrate, are more active around neutral pH [10], whereas PPO activity in kiwi is maximal at pH 8.0 [24]. The pH optimum of grape and olive PPOs depends on the cultivar, ranging from 3.5 to 7.3 and from 4.5 to 7.0, respectively [10,25,26,27]. Concerning herbaceous crops, potato and wheat PPOs show two pH optima (4.5 and 5.0 in potato; 5.3 and 6.9 in wheat), due to the presence of two ionization states of the enzyme–substrate complex and the existence of two different acid dissociation constants (pKs) [28,29]. In sweet potato, two different PPO isoforms exhibit different pH optima, one at pH 5.4 and the other at 6.7 [30]. Lettuce PPOs show a broad pH optimum, from 5.0 to 8.0 [31], whereas spinach PPOs have pH optimum around 8.0 [32]. Finally, eggplant has pH optima of 5.5 and 7.5 for diphenolase and monophenolase activity, respectively [33].

Temperature is another important factor significantly affecting the catalytic activity of PPO, as it influences the solubility of oxygen and may lead to enzyme denaturation [18,19,20,21,22,23]. The optimal PPO temperature range varies for different plant sources: 25–35 °C in lettuce, 25–45 °C in grape and 30–50 °C in olive [20,25,26,27,31,34]. PPOs from strawberry and cucumber show maximum activity at 50 °C [35,36]. Grape PPOs show an activity reduction of approximately 50% after 20 min at 65 °C, and a complete inactivation after 15 min at 75 °C [20]. At 60 °C, PPOs from apple have a half-life of 30 min [22]. PPOs from mango require more than 15 min at 80 °C for 50% loss of activity [37]. Cocoa beans and lettuce PPOs are inactivated after 5 min at 80 and 90 °C, respectively [31,32,33,34,35,36,37,38]. Thermal stability of olive PPOs is cultivar-dependent. Indeed, about 50% reduction of the initial activity was observed in cv. Donat and Gemlik after 60 min at 40 °C [26]. The same treatment was associated with 65% reduction of PPO activity in cv. Coratina [34]. The type of phenolic substrate can also affect PPO thermal stability [19,20,21,22]. In addition, sucrose and salts in the reaction environment act as protective agents against thermal denaturation [39].

### 2.2. Enzymatic Browning in Crops and Food Processing

Browning negatively affects the commercial value of many agricultural productions, including apple [40], banana [41], cucumber [36], grape [42], mango [37], pear [43], peach, apricot [21], eggplant [44], lettuce [31], potato [28] and cereals [45,46]. In a few cases, it is associated with the formation of flavour compounds positively influencing the food processing quality, for instance in the production of black tea [47], coffee [48] and cocoa [38].

The quali-quantitative composition of crop PPO phenolic substrates is variable in different crop species, resulting in different browning intensities. Catechin is the major phenolic substrate in grapes and tea [47,48,49], chlorogenic acid is abundant in apple [50], potato [28], sunflower [51], sweet potato [30,31,32,33,34,35,36,37,38,39] and eggplant [52], and ferulic acid is mainly present in wheat [53]. Finally, catechin, epicatechin and caffeic acid derivatives are common substrates of several fruit PPOs [54].

Must browning can originate from enzymatic and non-enzymatic oxidation, both resulting in the formation of *o*-quinones [1]. Overall, procyanidins and monomeric catechins are associated with higher browning degree than other phenolics [55]. Enzymatic browning depends on tyrosinases and laccases, which are produced from grape berries and moulds, respectively [1], and is associated with the content of hydroxycinnamates such as caffeoyltartaric acid (caftaric acid) and *p*-coumaroyltartaric acid (coutaric acid). In addition, peroxidases (PODs) can enhance the degradation of phenols when coexisting with PPOs [56]. Non-enzymatic oxidation is due to the conversion of polyphenols such as (+)-catechin/(−)-epicatechin, gallic acid, and caffeic acid, which are the most readily oxidized wine constituents [1].

Both PPO and POD enzymatic activities play a key role in determining the phenolic profile of olive oil. PPO activity is largely present in the olive mesocarp and is relatively constant after complete fruit ripening [57,58]. In contrast, peroxidase activity is mainly located in the seed. An increase in PPO activity has been observed in response to abiotic stresses [57,58,59]. Endogenous PPOs and PODs display their maximal activity mostly during the crushing and malaxation steps of the olive oil extraction process [60]. Considering that olive PPOs have optimal activity at 50 °C and lowest stability at 40 °C, the malaxation temperature is a crucial factor influencing the phenolic content of olive oils [34].

In wheat, PPOs have been reported to be responsible for browning of whole meal flour [61], dough [45,46,47,48,49,50,51,52,53,54,55,56,57,58,59,60,61,62] and several types of end-products such as Chinese steamed bread [63], Middle East flat breads [64], noodles [65,66] and the Italian Castelvetrano durum wheat bread [46,47,48,49,50,51,52,53,54,55,56,57,58,59,60,61,62,63,64,65,66,67]. PPOs are closely associated with the external layers of the caryopsis constituting the bran. Therefore, the distribution of PPOs in millstreams is closely related to the effectiveness of the milling procedure in removing the bran coat, and enzymatic browning is more evident in whole-meal flour and derived end-products [68]. At least 90% of the PPOs are removed with a flour yield over 70% (i.e., the conventional extraction rate for refined flour) [68].

In pasta, the yellow colour is related to the carotenoid content and the level of lypoxigenase activity of semolina. The red component is instead the result of Maillard reaction during drying. Finally, brownness depends on phenolics and PPOs. Interestingly, durum wheat cultivars have significantly lower PPO activity than cultivars of hexaploid wheat [69]. Among the pasta-making steps, dough formation is potentially the most suitable to enzymatic browning, as it facilitates the interaction between enzyme and substrates [70]. Today, the response of consumers to the browning of pasta is changing, due to the latest introduction of new pasta types obtained with whole meal flour or other cereals believed to be healthier [71,72].

### 2.3. Technological Strategies for Browning Prevention

Several practices can be followed by the food industry to prevent or limit enzymatic browning, including the use of reaction inhibitors, physical treatments and the use of by-product extracts and modified atmosphere. All these methods can be used alone or in combination [73]. Differently acting chemical inhibitors, such as reducing (ascorbic acid and sulphites), chelating (ethylenediaminetetraacetate), acid (citric and malic), and complexing compounds (cyclodextrins), inhibit melanin formation by preventing the accumulation of *o*-quinones, or by forming stable colourless products [10,73,74,75,76,77,78,79]. Sodium sulphite, bisulphite, and metabisulphite were extensively used in the fruit and vegetable processing industry, due to their low price [73,74,75,76,77,78,79,80]. However, their commercialization is banned in several Countries [73,74,75,76,77,78,79,80,81] because of adverse effects on health [82]. As alternatives, ascorbic acid and 4-hexylresorcinol (4-HR; 4-hexyl-1,3-benzenediol) are frequently used. In addition, cyclodextrins have a promising anti-browning effect, as shown for potato and in fruit juices [83,84,85]. A recent study pointed out that β-cyclodextrin binds non-covalently to PPOs better than other polysaccharides and has a relevant inhibiting ability under optimum conditions [84].

Physical treatments such as heat, dehydration, irradiation and high pressure are another strategy to minimize browning [77]. Blanching treatments by steam, hot water, acid/salt water solutions, and microwave are also largely used [86,87,88,89,90,91,92,93]. However, to balance the degree of inactivation of PPOs and the impact on the quality of end-products, time and temperature of blanching should be carefully set up [94].

Microwaved extracts obtained from by-products of the agro-industry, such as thinned fruits, represent another tool for the prevention of enzymatic browning [95]. Indeed, the microwave treatment induces the formation of Maillard reaction products (MRPs), associated with antioxidant activity. A solution of 2% extract was reported to inhibit PPOs in minimally processed peaches for eight days of storage [95]. Finally, modified atmosphere packaging with low permeability films is another effective way for extending shelf-life of processed fruit and vegetables, as oxygen is needed to trigger browning.

## 3. Physiological Roles of PPOs

PPOs have been largely investigated in relation to the commercial importance of browning. However, their role in plant physiology is less clear. Several studies report a positive correlation between PPO expression and resistance/tolerance to biotic stresses. For instance, potato varieties displaying enhanced PPO activity also exhibited higher tolerance to soft rot disease caused by the bacteria *Pectobacterium atrosepticum*, *Pectobacterium carotovorum* subsp. *brasiliensis* and *Dickeya* spp. [96]. Similarly, PPO expression could be used as a biochemical marker to predict the outcome of the interaction between different tomato genotypes and the pathogens *Ralstonia solanacearum* and *Xanthomonas axonopodis* pv. *vesicatoria*, causing bacterial wilt and bacterial leaf spot disease, respectively [97,98]. Concerning interactions with fungi, high and rapid accumulation of localized levels of PPOs were detected in pearl millet genotypes resistant to *Sclerospora graminicola*, the causal agent of downy mildew; conversely, susceptible cultivars failed to accumulate PPO even after a considerable time [99]. Likewise, rapid and localized overexpression of multiple forms of PPOs was induced in resistant wheat and chickpea genotypes challenged with *Fusarium graminearum* and *Fusarium oxysporum* f. sp. *ciceri*, respectively [100,101,102]. In plant–herbivore interactions, higher PPO contents were associated with increased potato tolerance to the Colorado beetle (*Leptinotarsa decemlineata*) [103].

Direct evidence showing the defensive role of PPOs came from a few functional studies. Li and Steffens [104] found that tomato plants overexpressing a potato PPO displayed enhanced resistance to *Pseudomonas syringae* pv. *tomato*, causal agent of the bacterial speck disease. Conversely, in plants transformed with antisense PPO cDNA, a significant reduction of PPO activity resulted in increased disease susceptibility [105]. In poplar, transgenic overexpression of PPOs enhanced resistance to herbivory by forest tent caterpillar (*Malacosoma disstria*) [106]. Possibly, the characterization of the *PPO* gene family in different plant species [107] and the availability of powerful tools for targeted gene inactivation and editing will provide further functional evidence on the defensive role of PPOs.

Many questions need to be answered on the physiology of PPOs. While it is generally assumed that PPOs play a role in defence responses to biotic stresses, the actual mechanism by which they exert their function is debated. In fact, they may act through: (1) direct toxicity of quinones; (2) reduced bioavailability and alkylation of cellular proteins to the pathogen; (3) cross-linking of quinones with protein or other phenolics, forming physical barriers; and (4) the production of reactive oxygen species (ROS), known to play an important role in defence signalling [103]. The involvement of PPOs in physiological circumstances not involving the disruption of cell compartmentation, resulting in the contact of PPOs with their vacuolar phenolic substrates, is seemingly counter-intuitive. However, it is known that PPO levels vary in response to drought or other forms of abiotic stresses. For example, the up-regulation of *PPO* genes was observed in tomato plants exposed to water stress, especially in the abscission zone of leaves and in leaf veins. However, control plants resulted less tolerant to water stress than genotypes in which PPO activity was suppressed [108]. Possibly, PPOs might have a role in photosynthesis as an oxygen buffer or interacting with the Mehler-peroxidase reaction. However, chloroplast phenolic substrates have not been identified [109,110].

## 4. Genomic and Genetic Aspects

The *PPO* gene family has been extensively studied in *Poaceae*, *Solanaceae* and in a few other species in which PPO activity affects economically important traits. Available genomic information for land plants indicates that the *PPO* gene family is extremely variable in size and structure, due to lineage-specific expansion and gene loss [107]. In fact, no putative *PPO* genes were identified in some species such as *Arabidopsis thaliana*. Different *PPO* homologs might be associated with very different regulatory patterns and expression profiles [111]. However, the factors influencing the expression of individual PPO members in plants are still unclear [112]. PPO proteins include two highly conserved copper-binding domains (CuA and CuB), each with three histidine residues, together forming a Cu^2+^ binding site, which has been widely used to isolate PPO cDNAs [107]. Typically, PPO proteins also encompass a N-terminal transit peptide, determining the import of PPOs into the thylakoid lumen, a dicopper centre, and a C-terminal region [107]. Although these three motifs are also generally conserved, rare variants have been identified [107]. Below, we report detailed information on genomic and genetic aspects of PPOs in several crop species.

### 4.1. Solanaceae

Six, seven and nine single-exon *PPO* genes were characterized in eggplant [112], tomato [113] and potato [114], respectively (Table S1). Mapping of these genes revealed that they are located on chromosome 8, thus suggesting an evolution based on tandem duplication events [114,115]

In tomato (*Solanum lycopersicum*) [115], *PPO* genes cluster in a 165-KB genomic region. Protein sequence conservation among family members, named PPOs A, A’, B, C, D, E and F (Table S1), ranges from 63% to 95%. Spatial and temporal differences in the expression of tomato PPO homologs were described [113]. PPO B is highly expressed in young leaves. Inflorescence also display high expression of PPO B together with PPO E and F. PPO A and C are specifically expressed in type I and type IV trichomes, whereas PPO D transcripts are only present in type VI trichomes. Promoter functional analysis mediated by GUS (β-Glucuronidase) fusion in transgenic tomato plants indicated that PPO F is responsive to abiotic and biotic challenges, thus suggesting a role in stress defence [116]. PPO B is localized predominantly to mitotic or apoptotic tissues and is induced by ethylene. Homology analysis of its promoter sequence revealed hits with genes involved in response to hormones, signal transduction, phenylpropanoid biosynthesis, photosynthesis, and the synthesis of fruit and seed proteins [113].

In potato (*Solanum tuberosum* L.), five PPOs were originally identified, by means of PCR amplification with primers designed on conserved PPO regions in the copper-binding domain [117], named *PPO-P1*, *PPO-P2*, *POT32*, *POT33* and *POT72*. A very high (>92%) protein identity was detected for the interspecific potato/tomato PPO pairs POT32/PPO D, POT33/PPO B, PPO-P2/PPO E, which might be indicative of functional conservation. A recent genome-wide search allowed the identification of nine *PPO*-like gene models, referred to as *StuPPO1*–*9* [114]. *StuPPO1*/*6* and *StuPPO2*/*3*/*4*/*5*/*7*/*8* form two genomic clusters on chromosome 8 of 47 and 144 KB, respectively, whereas *StuPPO9* is located on chromosome 2. Nucleotide similarities suggest that *StuPPO1*–*4* are allelic variants of *PPO-P1*/P2, *POT32*, *POT33* and *POT72*, respectively. The highest PPO activity occurred in roots, stolons, tuber buds and undeveloped flowers. Moderate levels of PPO activity were observed in young leaves, mature tubers, opened flowers, while low PPO activity was present in mature leaves and stems [117]. *PPO* genes did not contribute equally to the total PPO protein content in tuber tissues, with *StuPPO2* playing a major role. Low PPO activity and low browning tubers were obtained when the expression of *StuPPO1* to *StuPPO4* were simultaneously suppressed by means of artificial microRNAs [114]. Recently, novel putative miRNAs of PPO have been identified in relation to stress resistance and brown spot of potato [118].

Based on nucleotide and protein similarity, the six eggplant (*Solanum melongena* L.) *Ppo* homologs were assigned to two structural classes defined as class A (*SmePPO1–3*) and class B (*SmePPO4–6*) [112]. A linkage map based on an interspecific *S. melongena* × *Solanum incanum* population allowed the identification of a cluster of five *PPO* homologs (*SmePPO1–5*) on linkage group 8 [119], spanning an interval of 5.1 cM. Expression studies revealed that eggplant PPO transcripts are present in tissues upon mechanical cutting or pathogen attack, and accumulate in the stem and fruits [112]. In more detail, *SmePPO1* expresses in all tissues, such as roots, young and mature leaves and pre- and post-anthesis flowers; *SmePPO2* predominantly expresses in root tissues; *SmePPO3* and *SmePPO4* express in all tissues except for the roots; and *SmePPO5* and *SmePPO6* display average expression in young leaves, sprouts and fruits.

**Figure 2:**
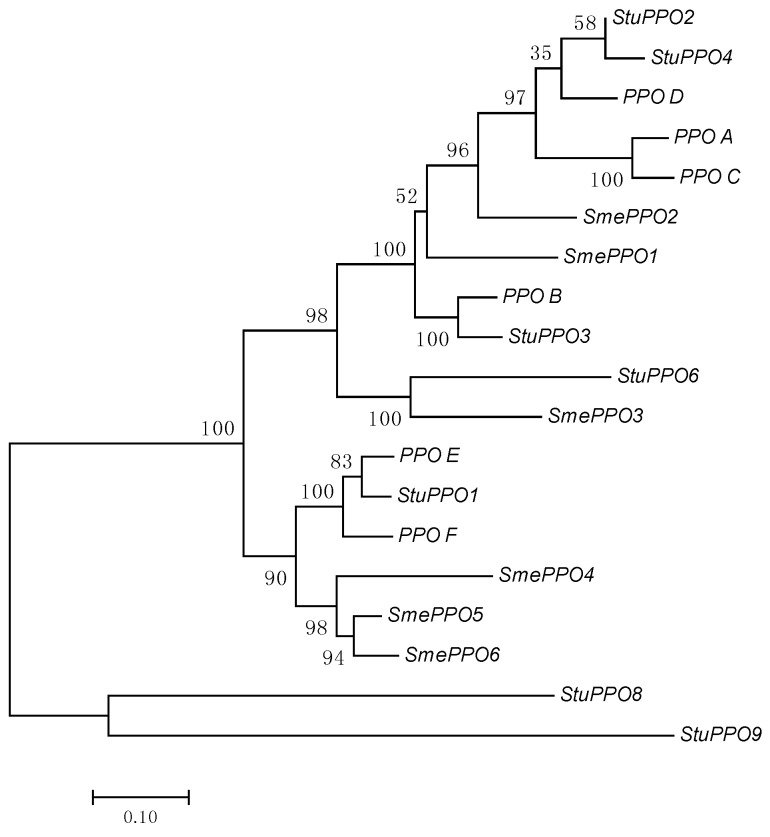
Evolutionary relationships among *Solanaceae* PPOs inferred by using the Maximum Likelihood method. Evolutionary analyses were conducted in MEGA7 (http://www.megasoftware.net/). Numbers below branches indicate 1000 bootstrap support values.

In this study, we carried out a phylogenetic analysis within PPO amino acid sequences from *Solanaceae*. The homologs *StuPPO5* and *StuPPO7* were excluded from the analysis as they do not show a complete sequence and possibly represent non-functional peptides [114]. The phylogenetic tree shown in Figure 2 is characterized by three clades. The first clade includes tomato *PPO A–D*, the eggplant class A (*SmePPO1–3*) and potato *StuPPO3/4*. Interestingly, except for *SmePPO3*, all the clade homologs for which the expression pattern is known (*SmePPO1*, *SmePPO2*, *StuPPO2*, and *PPO A*, *C*, and *D*) are transcribed in roots. The second clade includes tomato *PPO E* and *F* and the eggplant class B (*SmePPO4–6*), associated with defence responses [112,113,114,115,116]. Notably, *StuPPO8* and *StuPPO9*, forming a phylogenetically distant clade, are the only *Solanaceae PPO* genes structurally characterized by introns [114].

### 4.2. Poaceae

Most cloned and characterized *PPO* genes in *Poaceae* refer to tetraploid durum wheat and exaploid common wheat, thus they occur as duplicate or triplicate sets. Most studies mapped wheat *PPO* genes on the group 2 of homoeologous chromosomes, however a few works localized *PPO* loci on chromosomes of the groups 3, 5, 6 and 7 [69,70,71,72,73,74,75,76,77,78,79,80,81,82,83,84,85,86,87,88,89,90,91,92,93,94,95,96,97,98,99,100,101,102,103,104,105,106,107,108,109,110,111,112,113,114,115,116,117,118,119,120]. The first chromosomal localization of *PPO* genes was achieved using chromosome 2A substitution lines and the N2DT2A nullisomic-tetrasomic line, displaying higher PPO activity than other cytogenetic lines [120]. Later, PPO activity was found to be associated with a major QTL on the chromosome arm 2AL, linked to the simple sequence repeat (SSR) marker *Xgwm294*, accounting for more than 80% genetic variation [121]. PPO activity was also associated with the chromosome arm 2BL, even though the phenotypic effect in this case resulted to be lower than its homoeologous region on chromosome 2AL [69,70,71,72,73,74,75,76,77,78,79,80,81,82,83,84,85,86,87,88,89,90,91,92,93,94,95,96,97,98,99,100,101,102,103,104,105,106,107,108,109,110,111,112,113,114,115,116,117,118,119,120,121,122]. Finally, a major QTL was detected on chromosome arm 2DL, which explained 23% of phenotypic PPO variation [69,70,71,72,73,74,75,76,77,78,79,80,81,82,83,84,85,86,87,88,89,90,91,92,93,94,95,96,97,98,99,100,101,102,103,104,105,106,107,108,109,110,111,112,113,114,115,116,117,118,119,120,121,122,123]. Several studies suggested that *PPO* genes on the homoeologous group 2 are arranged in two paralogous families, named *Ppo-1* (*Ppo-A1*, *Ppo-B1* and *Ppo-D1*) and *Ppo-2* (*Ppo-A2*, *Ppo-B2* and *Ppo-D2*). *Ppo-A2*, *Ppo-B2* and *Ppo-D2* are located 8.9, 11.4 and 10.7 cM proximal to the respective paralogs of the *Ppo-1* family [124,125,126,127,128].

The three homoeologous genes forming the *Ppo-1* family display a very similar structure, as they consist in three exons and two introns. Among these genes, an insertion of 191-bp in the second intron of the *Ppo-A1* gene represents the most important nucleotide variation. In contrast, *Ppo-2* genes differ in structure, as *Ppo-2A* and *Ppo-2D* display one intron, whereas *Ppo-B2* displays two [125,126,127]. Interestingly, wheat *PPO* genes are among the few examples of homoeologous containing introns, shown to determine alternative splicing of premRNAs, in turn influencing the level of PPO activity [128,129,130].

Based on the *Ppo-A1* gene sequence, three functional markers (*PPO18*, *PPO33* and *MG18*) were developed to identify allelic variants underlying variation of PPO activity (Table S1) [45,130,131,132]. The functional marker *MG08* was developed for *Ppo-B1* and detected four allelic variants resulting in different PPO activity [128]. Finally, two dominant functional markers, *PPO16* and *PPO29*, mapped on the chromosome arm 2DL, were able to discriminate genotypes with low and high PPO activity [132].

Both *Ppo-1* and *Ppo-2* genes are expressed in developing kernels and affect PPO activity. However, they show variable expression levels. Specifically, *Ppo-2* genes contribute to over 70% of PPO activity, and *PPO* genes on chromosome 2A generate nearly 90% of the *PPO* transcripts [124,126].

In barley (2*n* = 2*x* = 14; genome HH), the *Ppo-1* and *Ppo-2* paralogs are closely linked on the long arm of chromosome 2H [133]. The respective orthologs were mapped on the chromosome arm 2HL^ch^ of wild barley *Hordeum chilense* (2*n* = 2*x* = 14; genome H^ch^H^ch^) and hexaploid *Tritordeum* (2*n* = 6*x* = 42; genome AABBH^ch^H^ch^) [134]. Association studies showed that both genes are involved in the phenol reaction of barley spikes and grains [133]. RT-PCR experiments revealed that *Ppo-1* was significantly expressed in awns and in hulls, with the highest content at two and three weeks after flowering, while *Ppo-2* was specifically expressed in caryopses two weeks after flowering [133].

*Oryza sativa* includes two subspecies, *japonica* and *indica*, that are distinguished for grain reaction to phenol treatment. Indeed, after exposure to 1%–2% aqueous phenol solution, *japonica* seeds do not show colour change (negative response), whereas seeds of the *indica* type and wild *Oryza* species assume a dark brown or black coloration (positive response). Particularly, discoloration was found to be controlled by a single Mendelian gene (*Phr1*) mapped on chromosome 4 in an interval flanked by the two markers S100 and S115. The cDNA sequence of *indica Phr1* showed high similarity with *PPO* genes and an amino acid sequence showing 68% identity with wheat *PPO* [135].

**Figure 3:**
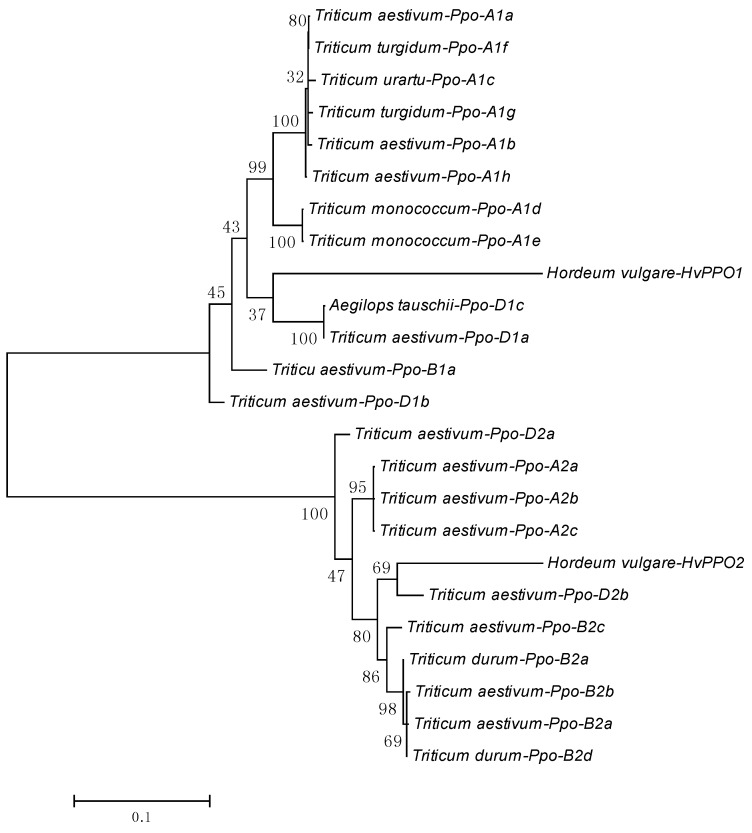
Evolutionary relationships between wheat and barley PPOs inferred by using the Maximum Likelihood method. Evolutionary analyses were conducted in MEGA7. Numbers below branches indicate 1000 bootstrap support values.

In Figure 3, we report the results of a phylogenetic analysis carried out using nucleotide sequences of wheat and barley PPOs. As expected, we found two clearly separated clades, corresponding to homologs of the *Ppo-1* and *Ppo-2* families.

### 4.3. Other Species

PPO activity in apricot (*Prunus armeniaca* L.), apple (*Malus domestica*) and banana (*Musa* ssp.) has been studied in relation to enzymatic browning during post-harvest stages [9,136,137]. In apricot, the *PA-PPO* gene was isolated from immature-green fruits, where it is highly expressed [138]. In an advanced stage of fruit development, PPO activity was detected, however no *PA-PPO* transcripts were detected in leaves, thus indicating high stability of the PPO proteins [138].

A recent study demonstrated that apple *PPO* genes are organized in clusters as observed for tomato [139]. Ten genes were annotated as *PPOs* and located on three distinct chromosomes (2, 5 and 10), including one, five and four elements, respectively. Interestingly, chromosomes 5 and 10 can be considered as homologous, due to the recent apple genome duplication [107,108,109,110,111,112,113,114,115,116,117,118,119,120,121,122,123,124,125,126,127,128,129,130,131,132,133,134,135,136,137,138,139]. The copper-binding domains of the apple pAPO5 and pMD-PPO2 proteins shared high conserved amino acids sequences with best-hit homologs in apricot (88% and 80%, respectively), grape berry (75% and 73%, respectively) and poplar (77% in both cases) [139,140]. Both *pAPO5* and *pMD-PPO2* genes are expressed in the early stages of fruit development.

High levels of PPO activity were detected in banana flesh throughout fruit growth and ripening. Four PPO cDNA were isolated from the banana fruit: BP01, BP011, BP034 and BP035 [41]. The BP01 genomic sequence was found to contain a 85-bp intron, whereas all the others were intronless. Expression analysis revealed a different expression pattern throughout the different stages of fruit development. BP01 was present in banana flesh early in development [41].

A recent work reported the cloning of a *PPO* gene in artichoke (*Cynara cardunculus* var. *scolymus* L.) [141]. In this species, browning may occur during early post-harvest stages and negatively affects quality. Based on degenerate primers, a *CsPPO* sequence was identified. *Cis*-acting elements were revealed in the promoter region, some of which are putatively involved in the response to light and wounds. The expression of *CsPPO* was significantly induced 48 h after wounding the capitula, even though the browning process had started earlier.

In walnut (*Juglans regia*), two *PPO* genes were characterized, named *JrPPO1* and *JrPPO*2, which share 80% and 71% of nucleotide and amino acid sequence identity. *JrPPO1* encodes a functional enzyme expressed primarily in the leaves, hulls, and flowers, whereas *JrPPO*2 is more expressed in callus tissues and in roots [8,9,10,11,12,13,14,15,16,17,18,19,20,21,22,23,24,25,26,27,28,29,30,31,32,33,34,35,36,37,38,39,40,41,42,43,44,45,46,47,48,49,50,51,52,53,54,55,56,57,58,59,60,61,62,63,64,65,66,67,68,69,70,71,72,73,74,75,76,77,78,79,80,81,82,83,84,85,86,87,88,89,90,91,92,93,94,95,96,97,98,99,100,101,102,103,104,105,106,107,108,109,110,111,112,113,114,115,116,117,118,119,120,121,122,123,124,125,126,127,128,129,130,131,132,133,134,135,136,137,138,139,140,141,142]. In walnut, the role of *PPO* genes has been related to pathogen resistance and the health-promoting activity of phenolic compounds as antioxidant and radical scavenging activities [8,9,10,11,12,13,14,15,16,17,18,19,20,21,22,23,24,25,26,27,28,29,30,31,32,33,34,35,36,37,38,39,40,41,42,43,44,45,46,47,48,49,50,51,52,53,54,55,56,57,58,59,60,61,62,63,64,65,66,67,68,69,70,71,72,73,74,75,76,77,78,79,80,81,82,83,84,85,86,87,88,89,90,91,92,93,94,95,96,97,98,99,100,101,102,103,104,105,106,107,108,109,110,111,112,113,114,115,116,117,118,119,120,121,122,123,124,125,126,127,128,129,130,131,132,133,134,135,136,137,138,139,140,141,142,143]. Very early work suggested that polyphenol oxidase activity produced a germicidal fluid that could inhibit bacterial growth [144,145].

## 5. Conclusions

The investigation of genes and mechanisms associated with PPO activity represents a pivotal challenge for food technology and plant breeding, considering the negative impact of enzymatic browning on the quality of primary productions and processed foods. Importantly, in the last few years, a large body of information is being accumulated on the genetic and genomic features of PPOs and their role in plant physiology. Possibly, our understanding on the role of PPO will benefit from recent advances in plant genomics and breakthrough discoveries in functional genetics, including targeted DNA modification by genome-editing.

## Supplementary Materials

Supplementary materials can be found at www.mdpi.com/1422-0067/18/2/377/s1.
